# The Steroid Veil: Complications in Identifying Lupus

**DOI:** 10.7759/cureus.76869

**Published:** 2025-01-03

**Authors:** Grace M Hingtgen, Silvija Milanovic, Jessica Portillo-Romero

**Affiliations:** 1 Department of Dermatology, University of Florida College of Medicine, Gainesville, USA; 2 Department of Internal Medicine, University of Florida College of Medicine, Gainesville, USA

**Keywords:** cutaneous lupus erythematosus, erythematous plaque, nikolsky sign, primary care, steroid, subacute cutaneous lupus erythematosus, sun-exposed skin

## Abstract

While topical steroids are an invaluable tool used for the treatment of pruritic rashes, they can also complicate attempts at a definitive diagnosis. The temporal association between steroid use and biopsy must be taken into account when investigating the underlying etiology of such rashes. In patients with a history of dermatologic disease with the onset of a new rash, management should include a biopsy, if deemed necessary for diagnosis, followed by a prescription of topical steroids for symptomatic treatment. A 63-year-old male with a history of psoriasis presented with a new-onset pruritic, erythematous-to-violaceous rash on sun-exposed areas. The final diagnosis of subacute cutaneous lupus erythematosus (SCLE) was delayed due to the use of topical steroids on cutaneous eruptions before presentation in the clinic for biopsy. Diagnosis of SCLE can be difficult, especially for primary care providers who do not see the initial presentation regularly, as cutaneous findings can be variable in presentation. However, recurrent eruptions in similar photo-distributed locations should alert providers of a potential underlying diagnosis and prompt referral for dermatologic evaluation should be suggested. Thorough personal and family history should be taken and photographs of the rash should be documented in the patient’s chart for future reference. Ultimately, biopsy is the gold standard diagnostic method for evaluating the etiology of new-onset rash. When there is suspicion of an underlying disease beyond idiopathic contact or irritant dermatitis, a biopsy should be considered as the next best step in management.

## Introduction

Cutaneous lupus erythematosus (CLE) is an autoimmune disorder with a wide variety of clinical manifestations, making it difficult to recognize at initial presentation. It presents across a spectrum, including acute cutaneous lupus erythematosus (ACLE), subacute cutaneous lupus erythematosus (SCLE), and chronic cutaneous lupus erythematosus (CCLE). SCLE typically presents as symmetrical, photo-distributed erythematous plaques and makes up 8% of all CLE cases [1,2]. The plaques seen in SCLE are typically categorized as either polycyclic annular or psoriasiform papulosquamous lesions; peripheral vesiculobullous lesions and crusts can also be seen surrounding the plaques [2]. Up to one-third of SCLE cases can be linked to a variety of medications, and these cases are often associated with positive anti-Sjögren’s-syndrome-related antigen A (SSA) antibodies [1,2]. Patients with drug-induced SCLE are often older and present with diffuse cutaneous involvement, including bullous and targetoid lesions [2]. Medications most often implicated include hydralazine, procainamide, isoniazid, methyldopa, quinidine, minocycline, and chlorpromazine [3]. It is diagnosed with skin biopsy and treated with topical corticosteroids or calcineurin inhibitors for limited disease and antimalarials for systemic disease [4]. Herein, we present a case that highlights the diagnostic challenges of SCLE and other differential diagnoses to consider when presenting with symmetrical, violaceous plaques in sun-exposed areas.

## Case presentation

**Figure 1:**
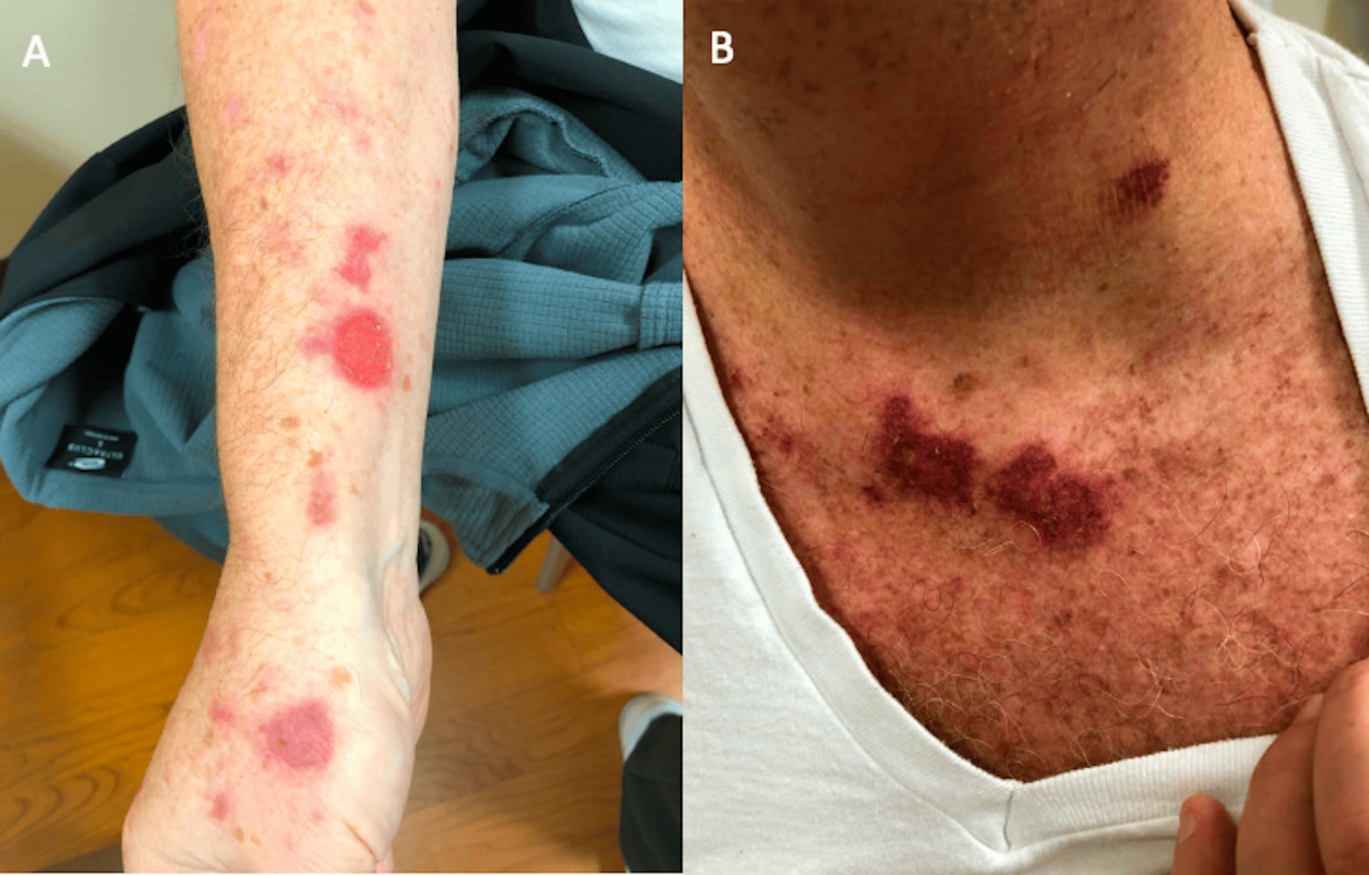
Patient’s first visit (week 13) shows several well-demarcated, somewhat annular, erythematous-to-violaceous papules and plaques with overlying scale and crust on the (A) right forearm, hand, and (B) chest. The crust is primarily localized to the peripheral borders, with small areas of surrounding erythema.

A 63-year-old Hispanic male with a past medical history of hypertension, hyperlipidemia, gout, and psoriasis controlled with atenolol, lisinopril, atorvastatin, allopurinol, moisturizers, and topical steroids presented to the clinic for a vesicular rash. Some of the vesicles ruptured, leaving erythematous, pruritic lesions, which the patient treated with Neosporin and cortisone cream, resulting in mild improvement. The rash subsequently spread to the face involving the left upper face and right lower vermillion lip. During this time, he remained afebrile with no other infectious symptoms. There was no recognizable inciting event or trigger. A similar rash appeared three months prior for which he used a topical cortisone cream with complete resolution after a few days. On physical exam, several well-demarcated plaques with overlying scale and crust were seen on the right hand, chest, lower neck, back, and lower legs (Figure 1). There were also a few intact bullae on erythematous bases and a positive Nikolsky sign, a clinical test in which lateral pressure on the skin induces separation of the epidermis from the dermis. Suspicion for pemphigus vulgaris was high due to the involvement of the oral mucosa and the presence of flaccid vesicles and bullae, along with other conditions such as mucous membrane pemphigoid and paraneoplastic pemphigus. The patient was referred to dermatology, where two punch biopsies were taken from the right volar hand and right calf. Pathology favored a resolving arthropod bite, and the patient was treated with mometasone.

Five months later, the patient presented for recurrence of pruritic rash in the same sun-exposed areas. At presentation, he had already completed several days of oral steroids prescribed from a walk-in clinic and the rash was beginning to resolve. The upper chest, neckline, right anterior thigh, ventral arms, and mid-central back were covered by several hyperpigmented, slightly erythematous macules and patches. He was concurrently experiencing a psoriasis flare as a few salmon-colored scaly papules, and plaques were noted on the anterior thigh and extensor elbows. A repeat punch biopsy was indicated to differentiate between suspected lichenoid drug eruption versus spongiotic dermatitis as the initial biopsy did not align with clinical suspicion and presentation. Pathology was consistent with post-inflammatory hyperpigmentation, which signified a resolving inflammatory pathology. No diagnosis was made at this time.

**Figure 2:**
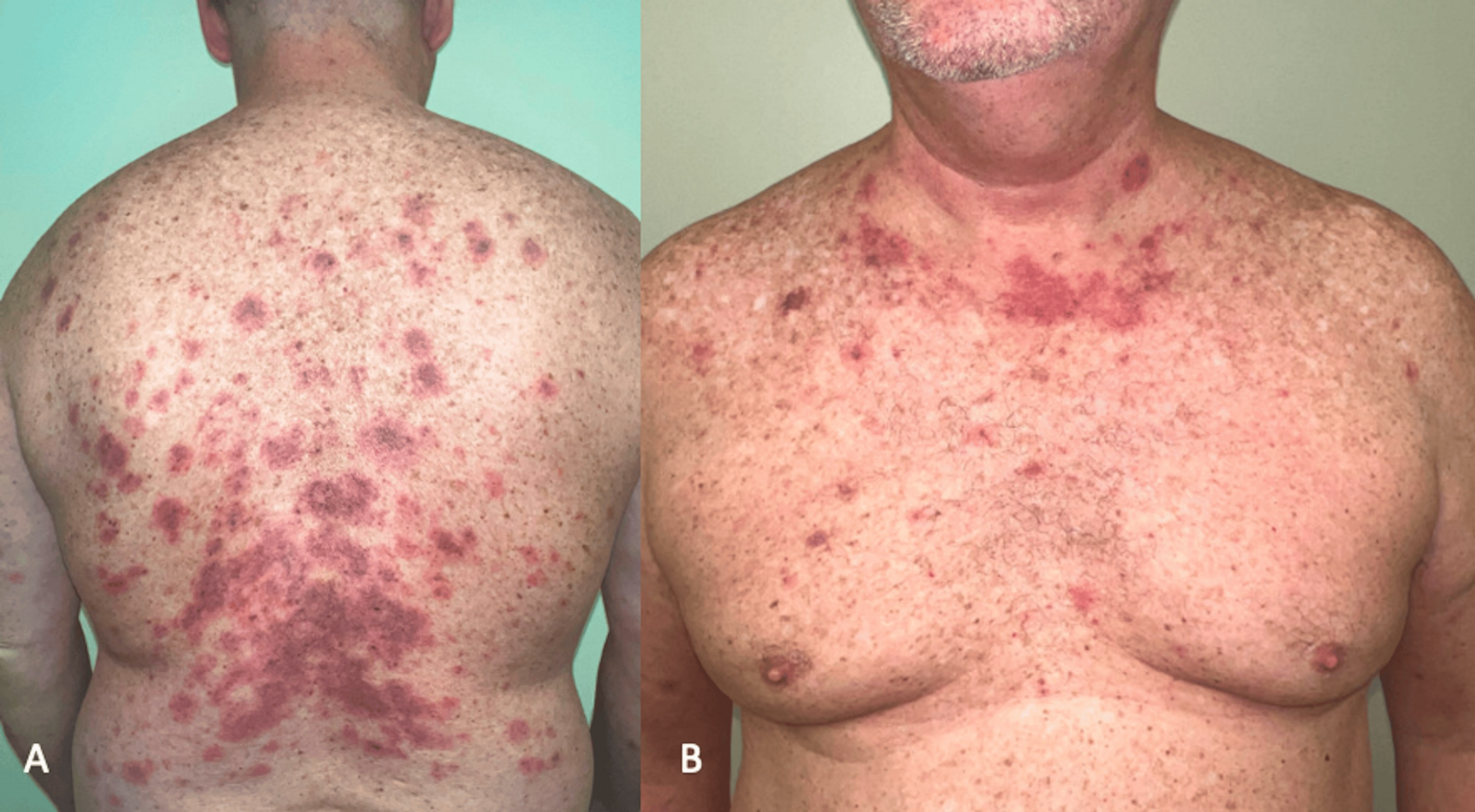
Patient’s follow-up visit (week 37) shows annular, violaceous patches and plaques with minimal secondary change located diffusely on the (A) back and (B) chest. Lesions are predominantly localized to the lower back where they appear as a deeper violaceous color. Again, there is some crust seen with an underlying base of erythema. The symmetrical distribution is helpful in narrowing down the differential diagnosis to an autoimmune or inflammatory dermatosis.

**Figure 3:**
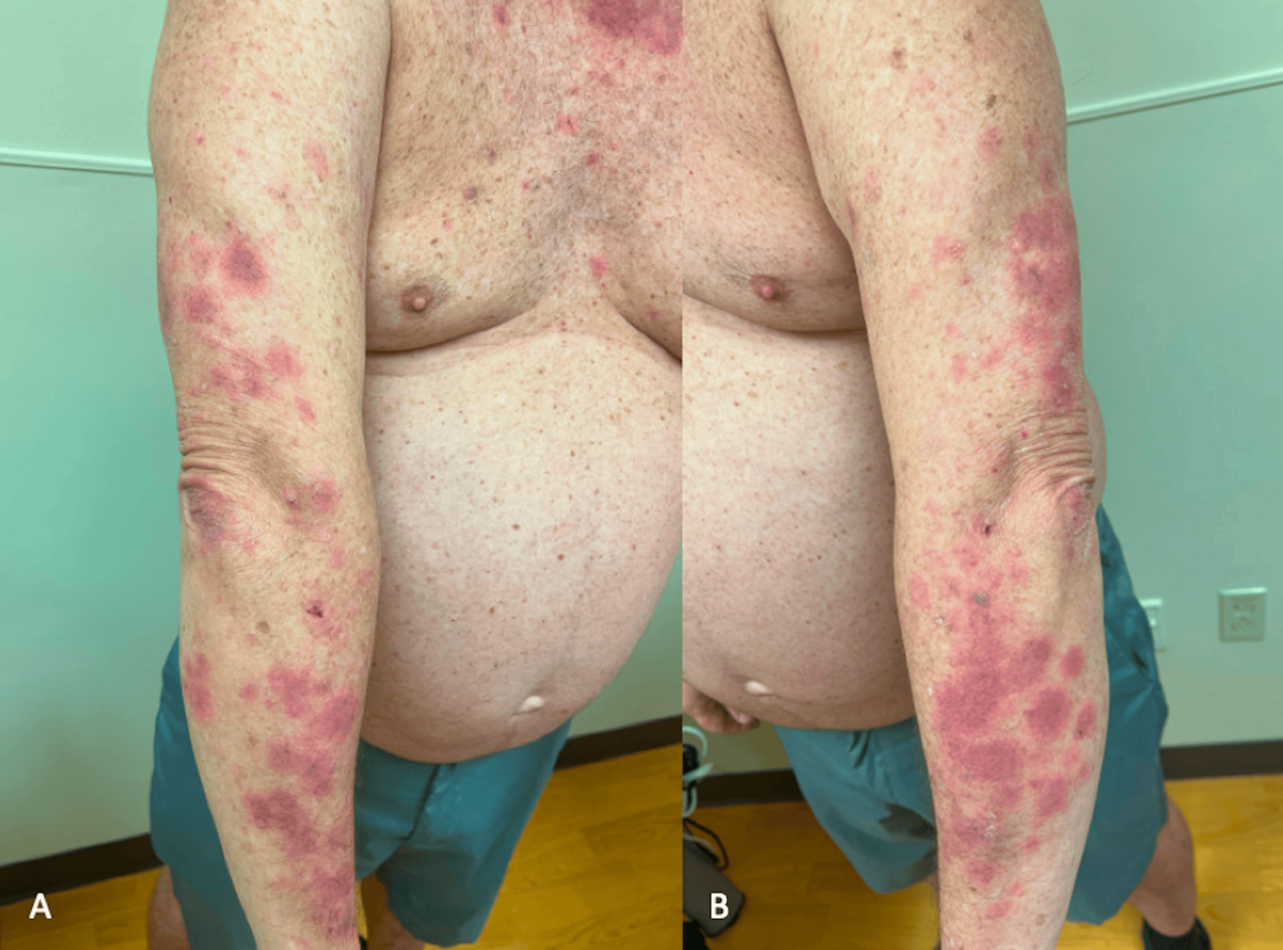
Follow-up after a 15-day course of prednisone shows symmetrical, targetoid papules and plaques with violaceous center and pink erythema peripherally on the bilateral arms with the absence of secondary changes or crusting. These findings are considered erythema multiforme-like and raised suspicion for Rowell syndrome; however, this diagnosis was ruled out due to a lack of supportive laboratory findings.

**Figure 4:**
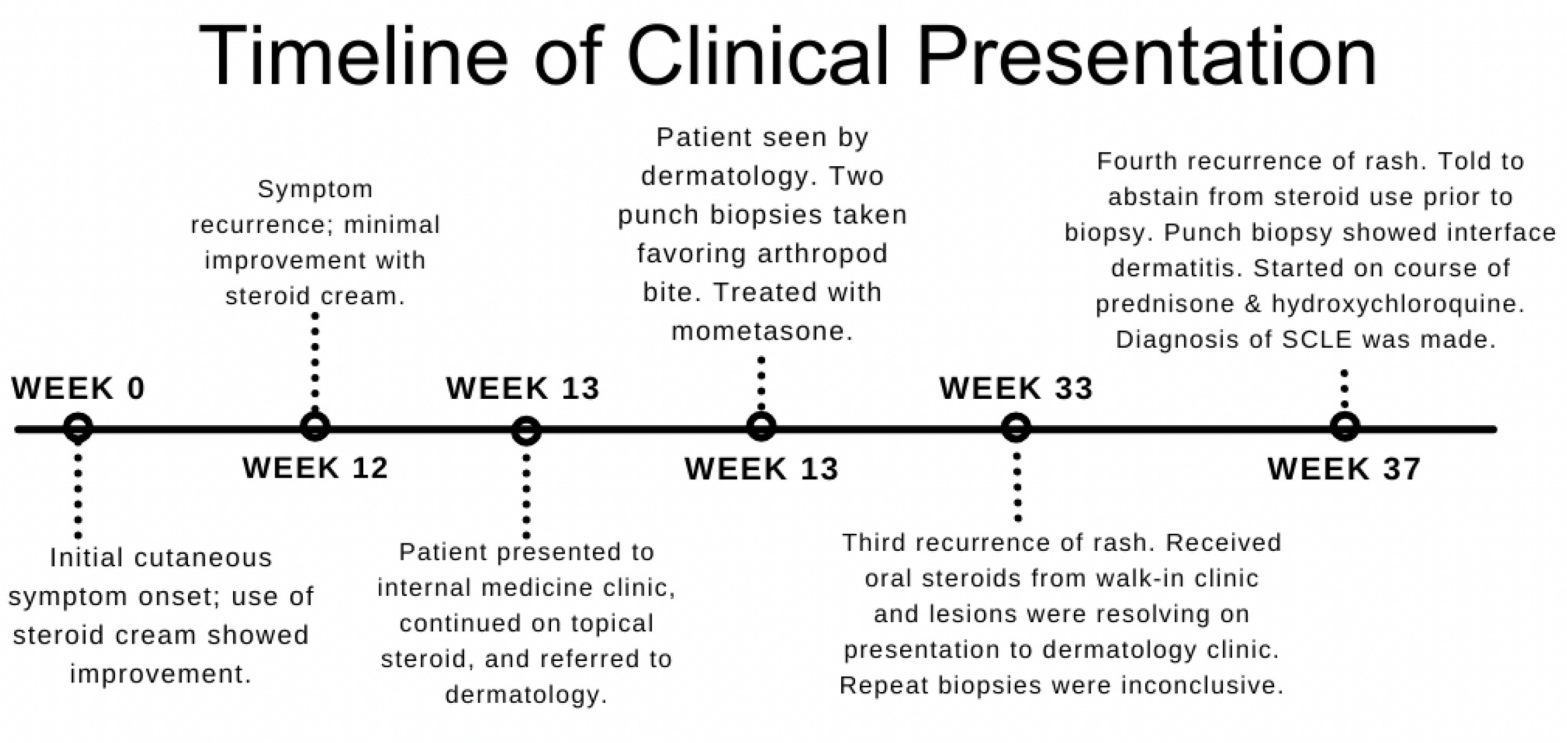
Timeline of clinical presentation and biopsies starting from initial symptom onset to final diagnosis.

One month later, the patient’s rash recurred. He was instructed not to take any steroids to allow for a representative biopsy in the clinic. Physical exam showed annular violaceous patches and plaques with minimal secondary change located diffusely on the trunk and extremities as well as erythematous plaques on the face (Figure 2 and Figure 3). Erythema multiforme-like SCLE (Rowell syndrome) was suspected based on the morphology and distribution of the rash. A punch biopsy was repeated with the goal of establishing a clear diagnosis, labs were drawn, and the patient was instructed to start a 15-day course of prednisone. Biopsy showed interface dermatitis with hyperkeratotic, acanthotic epidermis, patchy interface vacuolar changes, numerous apoptotic keratinocytes, moderate perivascular and interstitial lymphocytic infiltrate with melanophages, and solar elastosis. These findings were consistent with a subacute inflammatory dermatosis. Laboratory values were significant for elevated C3 and C4 (Table 1). Other autoimmune labs were negative including antinuclear antibodies (ANA), SSA, Sjögren’s-syndrome-related antigen B (SSB), rheumatoid factor (RF), and anticardiolipin IgG. Anti-histone antibodies were not assessed. The patient was subsequently diagnosed with SCLE, and hydroxychloroquine 200 mg twice daily was started. Following prednisone taper, the rash resolved with residual post-inflammatory hyperpigmentation. Of note, it was discovered that the patient’s brother had also been diagnosed with lupus of unknown characterization several years prior. A timeline of clinical presentation is outlined in Figure 4.

**Table 1:** Autoimmune laboratory values.

Autoimmune Markers	Value
Anticardiolipin IgG ≤20.0 GPLU/ML	<9.4
Antinuclear antibodies, IFA	<1:80 negative
Sjogren’s anti-SS-A 0.0 - 0.9 AI	<0.2
Sjogren’s anti-SS-B 0.0 - 0.9 AI	<0.2
C3 Complement 82 - 167 mg/dL	188 high
Complement C4, serum 12 - 38 mg/dL	40 high
Rheumatoid factor 0.0 - 14.0 IU/ML	<10.0

## Discussion

This case illustrates the diverse presentations of SCLE, often termed the great masquerader due to its ubiquitous presentation at the onset. Diagnostic challenges are heightened in patients with significant dermatologic histories, where assumed flares of known conditions may lead to delayed recognition of new underlying diseases, a phenomenon attributable to availability bias. Physicians must remain vigilant to avoid such cognitive pitfalls.

SCLE typically presents as symmetrical, photo-distributed erythematous plaques. The two primary variants include the polycyclic annular type and the psoriasiform papulosquamous type, the latter often mimicking psoriasis [2]. In this case, the patient exhibited recurrent rashes in photo-distributed areas; however, the lesions varied slightly in appearance at each presentation due to confounding factors (i.e., steroid usage). The rash initially appeared vesicular with well-demarcated scaly plaques and crusts. Features like positive Nikolsky sign, flaccid vesicles, and oral mucosal involvement were suggestive of pemphigus vulgaris. As oral involvement in SCLE is rare, it is often not considered in the differential diagnosis for mucosal blisters [5,6]. However, SCLE-related oral lesions can present with atrophic circular erythematous plaques [2].

Initial pathology suggested a resolving arthropod bite as immunofluorescence was negative. On follow-up, after oral steroid treatment, the rash resolved into hyperpigmented, slightly erythematous macules and patches, suggestive of lichenoid drug eruption or spongiotic dermatitis. Lichenoid drug eruptions, while less common than the classic morbilliform drug eruption or urticarial reactions, should be considered in a new-onset symmetric, erythematous, papular rash in patients recently started on antimalarials, beta-blockers, angiotensin-converting-enzyme inhibitors, biologics, or immune checkpoint inhibitors [7]. However, lichenoid drug eruption has rarely been associated with beta-blockers and the average latency from drug initiation to symptom onset is 15.7 weeks [8]. Lack of crusting, blistering, and significant pruritus made spongiotic dermatoses such as bullous pemphigoid less likely.

On the third recurrence, the patient presented with annular violaceous patches and plaques, morphology, and distribution suggestive of Rowell syndrome. Rowell syndrome is characterized by a combination of CTE and erythema multiforme-like lesions that are positive for ANA, SSA, SSB, and RF [9]. However, ANA, SSA, SSB, and RF antibodies were negative making Rowell less likely. To capture the true inflammatory histopathological features, steroids were held until the patient could be seen for a biopsy. It had been six weeks since the last use of topical steroids. There is no standard duration for which steroids should be held; however, it is ideal for the site to be steroid-free for at least one week or as long as possible. By temporarily delaying treatment, accurate diagnosis could be obtained. Pathology showed interface dermatitis favoring SCLE, ruling out drug eruption. In SCLE, the interface dermatitis is intense, with many cytoid bodies. The lymphocytic infiltrate is superficial and predominantly perivascular [2].

The primary aim in treatment for SCLE is twofold. Therapies are intended to resolve current cutaneous symptoms and prevent future occurrences. First-line therapy includes topical steroids as well as strict sun protection or sun avoidance [1]. Medium- to high-potency topical steroids are favored and can be applied to affected areas twice daily [1,2]. They may be used for two weeks at a time or until symptom resolution, followed by a one to two-week hiatus from steroid use in order to minimize the change of side effects (i.e., telangiectasia, atrophy, and striae) [1,2]. In severely symptomatic patients who have already initiated topical steroids before biopsy, it may be helpful to preemptively treat through the current flare. Patient education should include a prompt return for biopsy upon subsequent rash recurrence without the use of steroids. While topical steroids are helpful, they are sometimes inadequate during serious flares. During such flares, a short course of systemic steroids may be used. Systemic steroids, however, should not be used routinely as the effects are temporary, and systemic dosing has a higher risk of undesirable side effects [1,2]. For skin involvement not controlled with photoprotection and topical steroids, antimalarial agents may also be considered [1,2]. Hydroxychloroquine is often used; however, patients will need to schedule regular eye exams to assess for retinal damage [2].

One-third of SCLE cases are drug-induced and associated with a range of medications including anti-hypertensives, statins, proton-pump inhibitors, and anti-tumor necrosis factor (TNF) agents. The patient’s only medication of concern was atenolol, which has rarely been associated with SCLE [10]. In addition, the patient has been on atenolol for over 30 years with no recent changes, making drug-induced etiology less likely. Notably, the patient’s family history of lupus suggests a potential genetic component that warrants exploration. Approximately 50% of SCLE patients also meet the criteria (mucocutaneous involvement, myalgias, arthritis, positive serology) for systemic lupus erythematosus (SLE), but rarely exhibit severe systemic symptoms such as lupus nephritis or vasculitis [11]. Adequate family history assessment is vital with recent studies showing 34% of patients with SCLE have a positive family history of SCLE or SLE [12]. This demonstrates the importance of longitudinal multidisciplinary care for these patients.

Ultimately, the diagnosis of SCLE involves several components including clinical presentation, time course, distribution, histopathologic findings, and laboratory studies. All of these together can be used to differentiate from other clinical etiologies, which may produce similar symptoms. Table 2 [1,2,13-15] provides an outline of differential diagnoses to consider when presented with symmetrical violaceous patches and plaques in sun-exposed areas.

**Table 2:** Differential diagnosis of symmetrical violaceous patches and plaques in sun-exposed areas. SCLE, subacute cutaneous lupus erythematosus; CLE, cutaneous lupus erythematosus; DIF, direct immunofluorescence; ANA, antinuclear antibodies; SSB, Sjögren’s-syndrome-related antigen B; SSA, Sjögren’s-syndrome-related antigen A

Diagnosis	Clinical Presentation	Typical Distribution	Histopathologic Findings	Laboratory Findings
SCLE	Annular or polycyclic erythematous plaques on the upper extremities, neck, or trunk; not typically pruritic	Symmetric, often sun-exposed	Perivascular and periadnexal lymphocytic infiltrate in the superficial and deep dermis with interface dermatitis; positive DIF for IgG or C3 at the dermo-epidermal junction	ANA (positive in 52-80% of SCLE patients), anti-Ro/SSA (positive in 70-90%), and anti-La/SSB (positive in 30-40%)
Rowell Syndrome	Erythema multiforme-like lesions in patients with a history of CLE	Symmetric, often sun-exposed with occasional involvement of palms and soles	Considered a variant of SCLE, therefore histologic findings are similar.	Speckled ANA, positive anti-Ro/SSA and anti-La/SSB antibodies, and positive RF
Bullous Pemphigoid	Significant pruritus; may have urticarial papules and plaques but is typically characterized by tense bullae	May be extensive or localized; usually symmetrical favoring flexural areas	Eosinophilic spongiosis with positive DIF demonstrating linear IgG1/4 and C3 along the basement membrane.	Positive anti-BP180 or anti-BP230 antibodies
Pemphigus Vulgaris	Painful mucosal ulceration with flaccid blisters, residual erosions, and overlying crust	Seborrheic distribution along the chest and upper back often with mucosal involvement	Acantholysis with blisters seen above the dermo-epidermal junction	Positive anti-desmoglein 1 and 3 antibodies
Arthropod Bite	Variably-sized erythematous papules with occasional vesicles or bullae; often associated with localized swelling, redness, pain, and pruritus.	May or may not be symmetrical or in sun-exposed areas depending on bite distribution	Wedge-shaped lymphocytic infiltrate with dermal edema; eosinophils are often seen and spongiosis may be present with vesicle formation	N/A
Lichenoid Drug Eruption	Violaceous, flat-topped papules or plaques, which are often very pruritic with pronounced desquamation; may have psoriasiform or eczematous morphology; lack of Wickham striae (to differentiate from traditional lichen planus)	Bilateral extremities with possible oral or genital involvement	Lichenoid interface dermatitis with lymphocytic infiltrate in the papillary dermis, focal parakeratosis, and prominent necrotic keratinocytes and other inflammatory cells	N/A

## Conclusions

This case contributes valuable insights into the diagnostic and therapeutic aspects of SCLE, especially with the absence of specific autoantibodies, prompting further inquiry into the spectrum of immune responses in SCLE. Additionally, it underscores the masking effect of steroids on the prompt diagnosis of SCLE, as steroid use can obscure the clinical picture, delaying accurate diagnosis and treatment. A thorough assessment and history, judicious use of diagnostic tools, and consideration of atypical presentations are vital in recognizing the complex presentation of autoimmune skin disorders such as SCLE in the primary care setting. We recommend avoidance of topical steroids for at least one week on biopsy locations when possible, but understand this may not always be possible in clinical practice. In cases where steroids have already been used, consider finding an area of involvement that the patient might not have reached or encourage prompt return for follow-up in the case of future rash recurrence for proper biopsy. Following an adequate biopsy, medium- to high-dose topical steroids should be prescribed as first-line therapy to be applied twice a day until the lesions resolve.

## References

[1] Walling HW, Sontheimer RD (2009). Cutaneous lupus erythematosus: issues in diagnosis and treatment. Am J Clin Dermatol.

[2] Vale EC, Garcia LC (2023). Cutaneous lupus erythematosus: a review of etiopathogenic, clinical, diagnostic and therapeutic aspects. An Bras Dermatol.

[3] Dalle Vedove C, Simon JC, Girolomoni G (2012). Drug-induced lupus erythematosus with emphasis on skin manifestations and the role of anti-TNFα agents. J Dtsch Dermatol Ges.

[4] Winkelmann RR, Kim GK, Del Rosso JQ (2013). Treatment of cutaneous lupus erythematosus: review and assessment of treatment benefits based on Oxford Centre for evidence-based medicine criteria. J Clin Aesthet Dermatol.

[5] García-Ríos P, Pecci-Lloret MP, Oñate-Sánchez RE (2022). Oral manifestations of systemic lupus erythematosus: a systematic review. Int J Environ Res Public Health.

[6] Siu A, Landon K, Ramos DM (2015). Differential diagnosis and management of oral ulcers. Semin Cutan Med Surg.

[7] Merk HF, Vanstreels L, Megahed M (2018). [Lichenoid drug reactions]. Hautarzt.

[8] Maul JT, Guillet C, Oschmann A (2023). Cutaneous lichenoid drug eruptions: a narrative review evaluating demographics, clinical features and culprit medications. J Eur Acad Dermatol Venereol.

[9] Gallo L, Megna M, Festa B, Stellato P, di Pinto R, Fabbrocini G, Ferrillo M (2020). Rowell syndrome: a diagnostic challenge. J Clin Aesthet Dermatol.

[10] McGuiness M, Frye RA, Deng JS (1997). Atenolol-induced lupus erythematosus. J Am Acad Dermatol.

[11] Okon LG, Werth VP (2013). Cutaneous lupus erythematosus: diagnosis and treatment. Best Pract Res Clin Rheumatol.

[12] Keum H, Brown LS, Chong BF (2022). Black patients with cutaneous lupus are associated with positive family history of cutaneous lupus and systemic lupus. Lupus Sci Med.

[13] Miyamoto D, Santi CG, Aoki V, Maruta CW (2019). Bullous pemphigoid. An Bras Dermatol.

[14] Tziotzios C, Lee JY, Brier T (2018). Lichen planus and lichenoid dermatoses: clinical overview and molecular basis. J Am Acad Dermatol.

[15] Melchionda V, Harman KE (2019). Pemphigus vulgaris and pemphigus foliaceus: an overview of the clinical presentation, investigations and management. Clin Exp Dermatol.

